# A systematic review and meta-analysis on the preventive behaviors in response to the COVID-19 pandemic among children and adolescents

**DOI:** 10.1186/s12889-022-13585-z

**Published:** 2022-06-15

**Authors:** Feifei Li, Wei Liang, Ryan E. Rhodes, Yanping Duan, Xiang Wang, Borui Shang, Yide Yang, Jiao Jiao, Min Yang, Rashmi Supriya, Julien S. Baker, Longyan Yi

**Affiliations:** 1 Center for Health and Exercise Science Research, Hong Kong Baptist University, Kowloon Tong, Hong Kong China; 2 Department of Sport, Physical Education and Health, Hong Kong Baptist University, Kowloon Tong, Hong Kong China; 3 School of Exercise Science, Physical and Health Education, University of Victoria, Victoria, Canada; 4 Department of Curriculum and Instruction, the Education University of Hong Kong, Ting Kok, Hong Kong China; 5 Department of Social Sciences, Hebei Sport University, Shijiazhuang, China; 6 Key Laboratory of Molecular Epidemiology of Hunan Province, School of Medicine, Hunan Normal University, Changsha, China; 7 Dr. Stephen Hui Research Centre for Physical Recreation and Wellness, Hong Kong Baptist University, Kowloon Tong, Hong Kong China; 8 China Institute of Sport and Health Science, Beijing Sport University, Beijing, China

**Keywords:** Preventive behaviors, COVID-19, Children and adolescents, Demographics, Psychosocial factors, Social and environmental factors, Review and meta-analysis

## Abstract

**Purpose:**

The purpose of this review was to synthesize the empirical evidence of relevant studies related to preventive behaviors in response to the COVID-19 pandemic among children and adolescents. Further to this, we aimed to identify the demographic, psychological, and social and environmental correlates of such behaviors.

**Methods:**

Following PRISMA guidelines, eligible literature was identified by searching seven databases (PsycINFO, PubMed, MEDLINE, EMBASE, Cochrane Library, PROSPERO registry platform, and ClinicalTrials.gov website) and reference list of included studies and relevant review papers from 1^st^ Jan 2020 to 28^th^ Feb 2021. The standardized mean difference and correlation coefficients *r* were extracted to estimate the effect sizes. Analyses were conducted using R software.

**Results:**

Of the 35,271 original papers, 23 eligible studies were included in the qualitative synthesis and all these studies were of moderate-to-high quality, of which 17 studies were further included into the quantitative analysis. Children and adolescents (6–20 yrs.) showed a poorer practice of COVID-19 preventive behaviors compared to younger adults (21–59 yrs.) with a small-to-medium effect size (SMD = -.25, 95%CI = -.41 to -.09). For the demographic correlates, children and adolescents’ COVID-19 preventive practice was found to be significantly associated with gender (*r* = .14, 95%CI = .10 to .18), while not with age (*r* = -.02, 95%CI = -.14 to .10). Narratively, knowledge was found to be consistently and significantly correlated. For the psychological correlates, small-to-medium overall effects were identified for the association with attitudes (*r* = .26, 95%CI = .21 to .31) and perceived severity (*r* = .16, 95%CI = .01 to .30). For the family and social correlates, a non-significant association was identified between family economic status and COVID-19 preventive behaviors (*r* = .004, 95%CI = -.12 to .12).

**Conclusions:**

Interventions and relevant policies of promoting children and adolescent’s preventive measures should be a priority. Further, empirical studies identifying the demographic, psychological, and family and social correlates of children and adolescents’ preventive behaviors are needed.

**Supplementary Information:**

The online version contains supplementary material available at 10.1186/s12889-022-13585-z.

## Introduction

The COVID-19 pandemic has continued for over two years, substantially influencing global societies. Up until the end of April 2022, there were more than 510 million confirmed cases and over 6.2 million deaths related to SARS-CoV-2 reported worldwide [1]. SARS-CoV-2 is primarily transmitted by respiratory droplets of infected or even asymptomatic people during direct contact with individuals and by contact routes [2–4]. Based on available evidence, WHO recommended droplet and contact precautions, which were consistent with national guidelines in most countries [5]. Preventive behaviors such as physical distancing, hand hygiene, and facemask wearing, have been advocated and proven to be effective in suppressing transmission of the virus and to flatten the pandemic curve [6]. Further, preventive behaviors are still needed following vaccination in order to enhance the suppressive effect (especially when transmission from one person to another following vaccination for COVID-19 is unknown) [7]. In addition, the omicron variant of SARS-CoV-2 is less responsive to two doses of vaccination, which results in substantial morbidity and mortality [8]. Therefore, preventive behaviors are still highly recommended to control the transmission of COVID-19.

Much research and public health messaging has been focused on adults and the role of children and adolescents in the prevalence and transmission of COVID-19 is still controversial. Children and adolescents were identified as being less susceptible to SARS-CoV-2 with mostly mild and asymptomatic cases compared with adults [9]. This might have underestimated the prevalence of COVID-19 among this population, as early routine testing and diagnosis was mainly restricted to symptomatic cases. The role of children and adolescents in the transmission of COVID-19 was also debated, particularly within the context of their threat to other vulnerable family populations [10]. In addition, children and adolescents were not the priority group to vaccinate in most countries.

Despite this early-stage lower focus, multisystem inflammatory syndrome in children, likely related to COVID-19 exposure or infection, has been increasingly reported and may lead to multi-organ failure [11]. Hence, practicing preventive behavior, as part of a comprehensive package of public health intervention, is important for children and adolescents.

A series of potential factors may determine and modify compliance with virus transmission preventive behaviors. Previous psychosocial theories, such as health belief model (HBM), the theory of planned behavior (TPB), health action process approach (HAPA), and ecological model, have proposed that individuals preventive behaviors could be affected by diverse demographic and psychosocial factors that should be targeted in the relevant behavioral promotion intervention and policy making [12]. For example, HBM suggested that individuals’ preventive behaviors are determined by their health beliefs (e.g., risk awareness, perceived susceptibility, and severity) [13, 14]. Both TPB and HAPA highlighted the intention-behavior relationship which provided a more comprehensive understanding for the individuals’ behavioral change process. TPB emphasized the antecedents of behavioral intention (i.e., motivational factors), such as social norms, attitudes, and perceived behavioral control, suggesting these factors affected the formation of behavioral intention that subsequently facilitated the behavioral change [15]. HAPA model focused on both motivational (e.g., risk perception, outcome expectancies, and action self-efficacy) and volitional factors (e.g., planning and action control) of behavioral change, highlighting the continuous process from intention formation to behavioral initiation and maintenance [16]. In addition to the above intrapersonal/individual factors, the ecological model suggested including the social and environmental (e.g., peers, parents, community environmental, and social policy) influence into consideration with respect to the promotion of health behavior [17]. An increasing group of evidence has supported the important role of these factors in initiating preventive behaviors in various populations, including children and adolescents [9]. However, as the characteristics of behaviors differ prominently across different age groups, distinguishing the correlates targeting children and adolescents is also required to the design of behavioral interventions and health policy decision-making in the fight against COVID-19 and future pandemics [18].

At present, there is limited summary evidence on both child and adolescents’ practice of preventive behaviors and relevant correlates during the initial stage of the COVID-19 pandemic. Therefore, the present study aimed to synthesize relevant evidence to explore the practice of COVID-19 preventive behaviors in children and adolescents compared with adult populations, and to identify the potential correlates (demographic, psychological, and social and environmental) of preventive behaviors among children and adolescents during the initial stage of the COVID-19 pandemic.

## Methods

The present systematic review and meta-analysis was undertaken following the Cochrane guidelines and GRADE approach [19], and the results were reported following the PRISMA statement and MOOSE guidelines [20, 21]. The study protocol has been prospectively registered on PROSPERO (CRD42021242062).

### Search strategy and study selection

Following the PICOS principles, the inclusion criteria were: (1) Population: studies including children and adolescents aged 6–20 yrs. were eligible according to the Colarusso’s recommendation (1992) [22]; (2) Intervention/exposure: studies targeting any preventive behaviors in response to the COVID-19 pandemic were included; (3) Comparison: not applicable for the observational studies. The baseline data of randomized controlled trials were used; (4) Outcomes of interest: the practice of preventive behaviors (e.g., physical distancing, face mask wearing, hand hygiene, and other preventive measures) in response to the COVID-19 pandemic, and the association of preventive practice with behavioral correlates (i.e., demographic, psychological, or family and social factors); and (5) Study types: cross-sectional, longitudinal cohort, randomized controlled trial, and experimental or quasi-experimental studies were eligible for inclusion, and commentaries. Pure qualitative assessments or case studies were not eligible. In addition, we only included full-text articles containing primary data that could be retrieved through online databases, library requests, or email correspondence with the authors.

According to a pre-defined literature search strategy (Additional file 1: Appendix 1), the following databases were searched electronically: PsycINFO, PubMed, MEDLINE (EBSCOhost used), EMBASE (Ovid platform used), and Cochrane Library. In addition, two databases were searched by hand: PROSPERO registry platform (COVID-19 theme) and ClinicalTrials.gov website. Finally, a hand search of the references of included papers and relevant systematic reviews was conducted. Since the COVID-19 pandemic was first announced by the WHO on 31 Dec 2019 [1], the time of the literature search was limited from 1^st^ Jan 2020 to 28^th^ Feb 2021. The literature search was restricted to human participants with no special requirements for language.

The primary search was conducted by two reviewers (JJ, FL) independently using Mendeley software. Following de-duplication, titles and abstracts were initially screened to exclude irrelevant studies. Then, the full texts of the remaining articles after initial screening were assessed for eligibility. Any disagreements between the two reviewers were resolved by consensus or confirmed by a third reviewer (WL).

### Study quality assessment

Two reviewers (WL/FL) independently evaluated the quality of the included studies using the National Institute of Health (NIH) quality assessment tool for observational cohort and cross-sectional studies (NIH, 2020) [23]. The NIH quality assessment tool used 14 measures of assessment, e.g. clarity of research questions, appropriateness of study population, sample size justification, quality of outcome measures, and accuracy of statistical analysis. The study quality was determined using four categories, “high = satisfying all assessed items”, “good = did not satisfy one item”, “moderate = did not satisfy two to four items”, and “poor = did not satisfy more than four items” [24]. Disagreement between the two reviewers were resolved by consensus or decided by a third reviewer (BS).

### Data extraction

Information of all eligible studies were extracted, including authors, regions, study design, date of data collection, sample size, participants’ demographics, preventive behavior(s), and the correlate measured. Two reviewers (WL/MY) independently extracted data and checked for errors mutually. Disagreements were resolved by consensus or by discussion with a third reviewer (FL). In our study, the outcomes included either a single preventive behavior (e.g., physical distancing, hand hygiene, facemask wearing) or combined preventive behaviors (e.g., practice aspect of knowledge, attitude, and practice [KAP] towards COVID-19). The practice score and/or percentage of adopting the preventive measures was extracted. Physical distancing referred to staying at home and away from crowded places, avoiding mass gatherings, and keeping certain space with each other, refraining from physical contacts especially with individuals who may sick. Hand hygiene referred to washing hands with soap, water, or alcohol-based hand rub lasting for a specified time. Referring to the potential determinates to preventive behaviors among children and adolescents, demographic, psychosocial, and family and social correlates were identified and categorized accordingly.

### Data synthesis and meta-analysis

Meta-analyses were implemented only if three studies at least provided effect sizes for the same parameter (only narrative analyses were conducted in case of not enough data). To compare the preventive practice across different age groups (study purpose 1), the sample size, mean score of preventive practice with standard deviation (SD), and number of adopting and non-adopting the preventive practice for each age group (6–20, 21–59, ≥ 60 yrs.) were extracted to calculate the standardized mean difference (SMD) and 95% confidence intervals (95%CI) [25]. For the investigation of behavioral correlates among children and adolescents (study purpose 2), the correlation coefficients (*r*) were extracted for the effect size estimates. Several studies used multivariate linear regression, the standardized regression coefficients (*β*) were converted to *r values* using a series of transformations [26]. Several studies only provided the OR, and the data was arithmetically converted to SMD or *r* using a spreadsheet where different formulas have been pre-inserted supporting for the automatic transformation of diverse effect sizes developed by DeCoster (2010) [27, 28]. All the data were converted to normally distributed Fisher’s *z* to calculate the pooled effect size and 95%CI using random effect models.

The percentage of total variation across the studies due to heterogeneity (Cochran’s *Q*-statistic) was used to calculate the *I*^2^ statistics, with 25%, 50%, and 75% indicating mild, moderate, and high degrees of heterogeneity, respectively [29]. Subgroup analyses were conducted if possible (e.g., the number of included effects is ≥ 10) to evaluate the robustness of the summary estimates to determine whether a particular study accounted for the heterogeneity [30, 31]. Sensitivity tests were conducted on study quality and study design. The publication bias was identified using the funnel plot, Egger’s regression test, and Fail-safe N approach [32]. All the analyses were conducted in *R* (R Core Team, 2013) and a *p* value of < 0.05 was considered statistically significant.

## Results

### Study characteristics

**Fig. 1:**
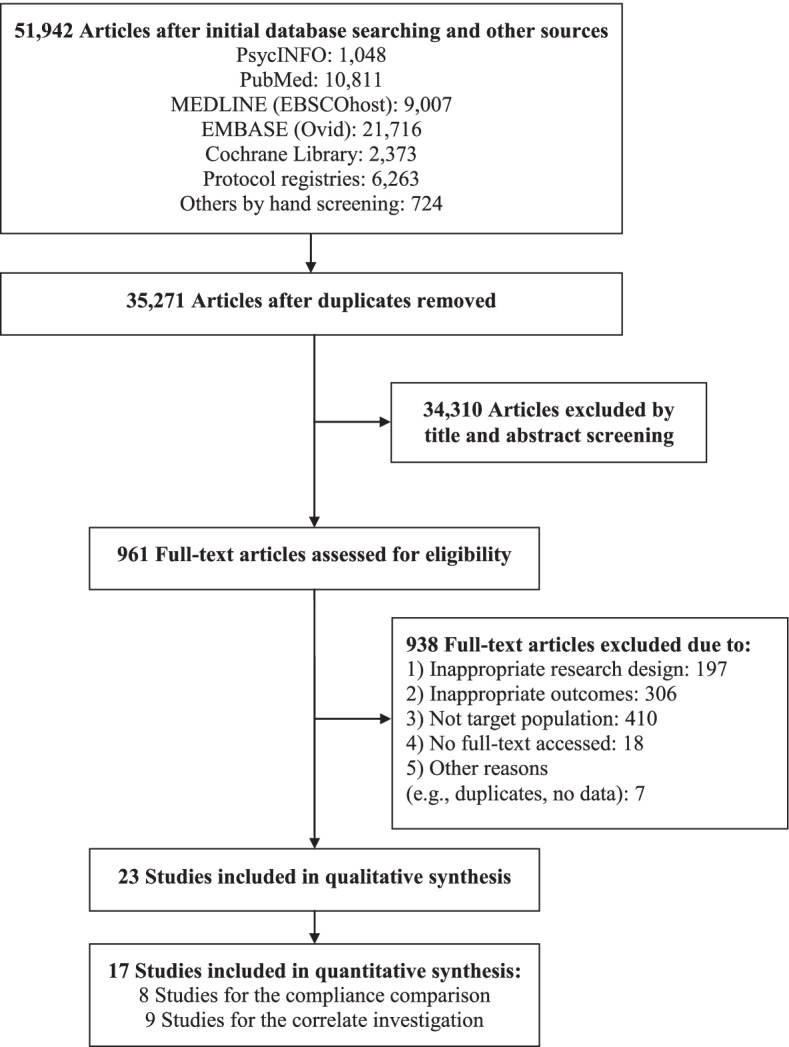
PRISMA flow chart outlining literature search process, inclusion and exclusion of studies

A total of 23 studies were identified and eligible for the present study as shown in Fig. 1. Among them, 17 studies which reported enough statistical information were extracted to the meta-analysis. A summary of study characteristics is presented in Table 1 and details of each included study are summarized in Additional file 2: Appendix 2. There were 11 studies that focused on the prevalence of preventive behavior containing children and adolescents (age 10–20 yrs.; n = 7,998) as a separate subgroup to compare with adults (age 21–59 and ≥ 60 yrs..; n = 62,172). Most of the studies were conducted in Asian countries (n = 10) and were observational studies with a cross-sectional design (n = 10). A total of four studies measured practice of KAP, six with multiple preventive behavior, and one focused only on each single behavior of physical distancing, hand hygiene, and facemask wearing. In addition, 12 studies that targeted only children and adolescents (age 6–20 yrs.; n = 19,663) to explore correlates of preventive behaviors during the COVID-19 pandemic. Five of the eligible studies were conducted in the Asian countries, with the remaining studies undertaken in countries of Europe (n = 4), North America (n = 2), and Africa (n = 1). All studies were observational studies, of which the majority used a cross-sectional design (n = 10). Multiple preventive behavior was the most frequently investigated outcomes (including practice of KAP; n = 8), and then physical distancing (n = 5).

**Table 1:** Summary of included studies

Characteristics	No. of studies	Percentages (%)
Total sample size	23	
Age group		
Mixed: children/adolescents and adults	11	
Only children and adolescents (6–20 yrs.)	12	
Region		
Asia	15	65.2
Europe	4	17.4
North America	2	8.7
Africa	2	8.7
Study design		
Cross-sectional	20	87.0
Longitudinal	3	13.0
Data collection period		
Jan – Apr 2020	16	69.6
May – Aug 2020	4	17.4
After Aug 2020	1	4.3
Not reported	2	8.7
Theoretical backdrop reported		
Health Belief Model	4	17.4
Self-determination Theory	2	8.7
Ecological Model	1	4.3
Integrated Cognitive Antisocial Potential Theory	1	4.3
Not reported	15	65.2
Preventive behaviors measured		
Multiple preventive behaviors	18	78.2
Single preventive behavior (physical distancing, hand hygiene, face mask wearing)	7	30.4
Individual demographics measured		
Age	7	58.3
Gender	12	100.0
Education level (e.g., primary/secondary/grade)	4	33.3
Others (e.g., ethnicity)	2	16.7
Psychosocial factors measured		
Attitude (e.g., perceived benefits/ barriers)	4	33.3
Knowledge	4	33.3
Risk perception (e.g., perceived susceptibility/severity)	3	25.0
Social interaction (e.g., trust, moral, norms)	4	33.3
Psychological well-being (e.g., depression, anxiety)	2	16.7
Others (e.g., intention, self-efficacy, motivation, personality)	4	33.3
Social environmental factors measured		
Family economic status	5	41.7
Residence (e.g., rural/urban)	3	25.0
Parents education background	4	33.3
Community setting and lockdown policy	4	33.3
Others (social media, parents birth location)	3	25.0
Study quality		
High	7	30.4
Good	12	52.2
Moderate	4	17.4
Poor	0	0

### Study quality assessment

The quality of selected studies was assessed by two authors independently (inter-rater agreement = 96%). All studies were of moderate-to-high quality. Particularly, 30.4% of studies were rated as high quality (*k* = 7), while 52.2% (*k* = 12) and 17.4% (*k* = 4) of studies were rated as good and moderate quality, respectively. Among studies with good and moderate quality, the major problem was the lack of reporting on sample size estimation (*k* = 14; 77.8%). In addition, three studies did not explicitly indicate the inclusion criteria for participants and another three did not report the reliability and validity of relevant measures (see Additional file 3: Appendix 3).

### Practice of preventive behaviors among children and adolescents

The majority of the included studies (18/23, 78.3%) targeted the practice of multiple preventive behaviors (e.g., hand hygiene, facemask wearing, physical distancing, covering coughs, house disinfection, intake vitamin C), where the percentage of children and adolescents adopting the preventive practice in response to the COVID-19 pandemic ranged from 16 to 94%. Seven studies targeted the specific preventive behavior of children and adolescents, indicating compliance rates of 76–89%, 60–88%, 31–87% for hand hygiene, facemask wearing, and physical distancing respectively.

**Fig. 2:**
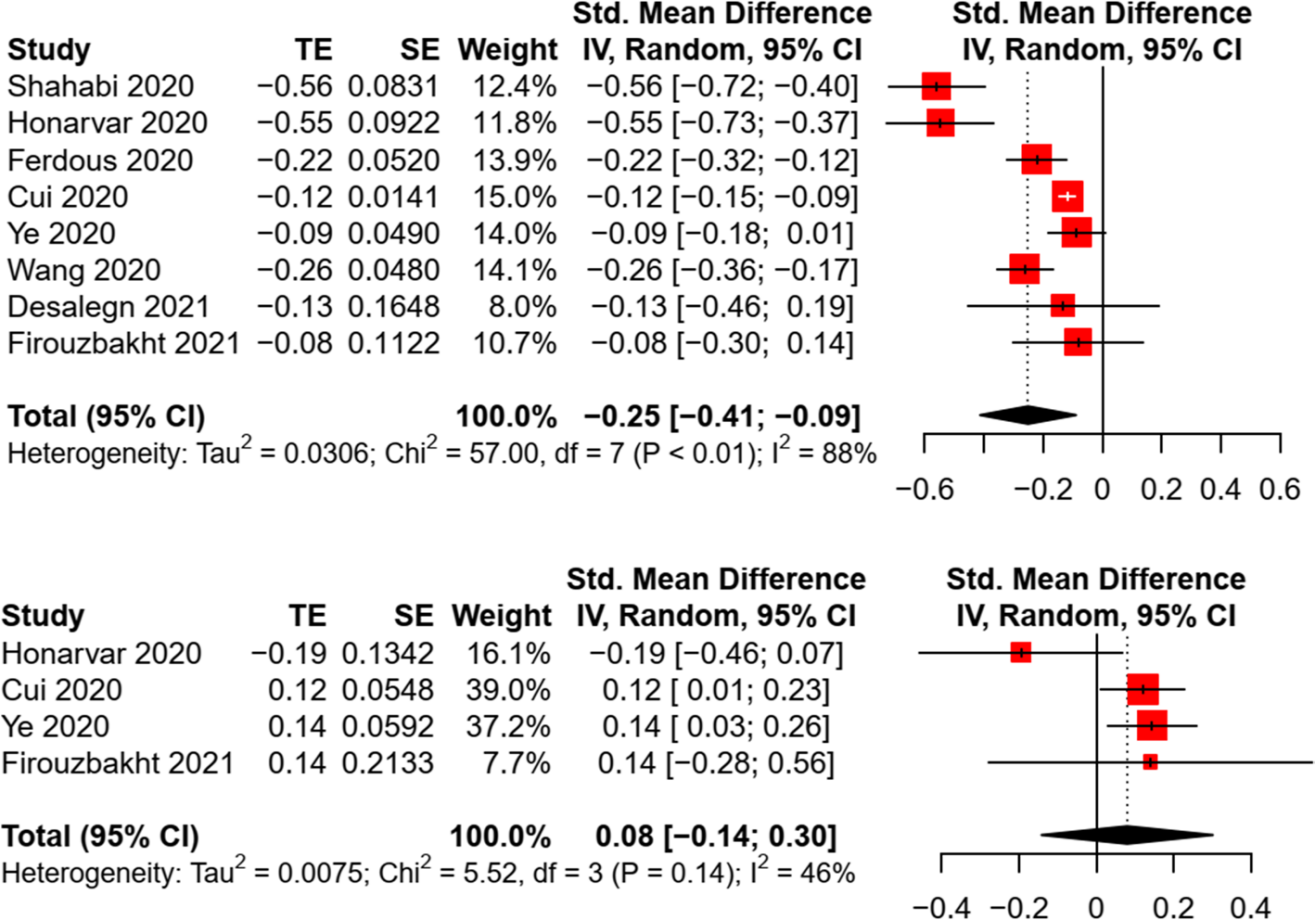
Random effect of the difference in preventive behaviors between children/adolescents and adults

When comparing the practice of COVID-19 preventive behaviors across different age groups, a pooled analysis of eight studies revealed a significant difference in preventive behaviors between children/adolescents and younger adults (21–59 yrs.), with a small-to-medium effect size (*k* = 8, *n* = 68,257, SMD = -0.25, *p* = 0.008; adults as reference) (Fig. 2). Heterogeneity was large between included studies (*Q* = 57.0, *I*^*2*^ = 88%, *p* < 0.001). Compared with older adults (≥ 60 yrs.), a non-significant difference in preventive behaviors was found in the meta-analysis (*k* = 4, *n* = 7,548, SMD = -0.08, *p* = 0.33; adults as reference). The effect sizes were comparatively homogenous across studies (*Q* = 5.52, *I*^*2*^ = 45.7%, *p* = 0.14). Sensitivity tests indicated a consistent result when excluding one article which was rated as moderate quality [33]. Per the inspection of funnel plots, Egger’s regression (all *p* < 0.05), and Fail-safe-N analyses, a non-significant risk of publication bias was identified for both comparisons (Additional file 4: Appendix 4).

### Demographic correlates of preventive behaviors in children and adolescents

**Fig. 3:**
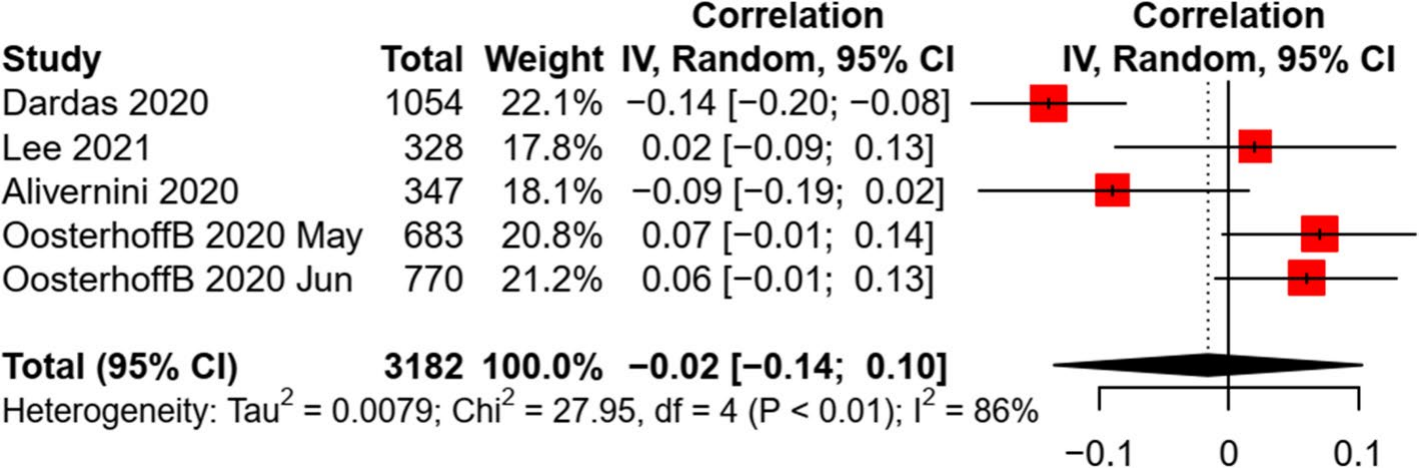
Random effect of age on preventive behaviors in children and adolescents

Age, gender, and education level were the most examined demographic factors. Of the 12 studies targeting only children and adolescents, seven studies examined the association of age with COVID-19 preventive behaviors. Three studies targeted multiple preventive behaviors and found no significant age-behavior correlation in children and adolescents [17, 34, 35], while similar results were also revealed in another four studies targeting hand hygiene or physical distancing [36–38]. A non-significant association between age and preventive practice was revealed in the meta-analysis (*k* = 5, *n* = 3,182, *r* = -0.02, *p* = 0.72) (see Fig. 3), with a large heterogeneity between studies (*Q* = 27.95, *I*^*2*^ = 86%, *P* < 0.001). Sensitivity test revealed a consistent finding when excluding a cohort study [39]. The funnel plot, Egger’s regression (*b* = 2.77, *p* = 0.68), and Fail-safe-N analyses indicated a non-significant publication bias (Additional file 4: Appendix 4).

**Fig. 4:**
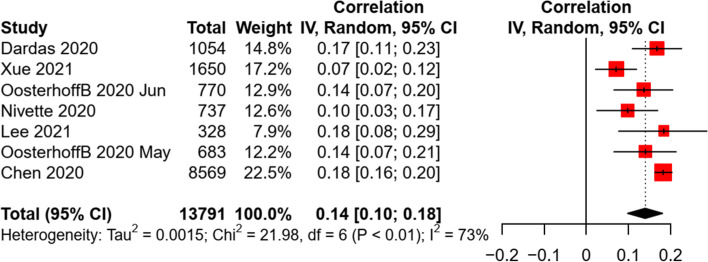
Random effect of gender on preventive behaviors in children and adolescents

All the 12 studies examined the difference in COVID-19 preventive behaviors across gender, among which seven studies were eligible to be included into the meta-analysis. A small and significant effect size was identified by synthesizing the data (*k* = 7, *n* = 13,791, *r* = 0.14, *p* < 0.001). A moderate heterogeneity of effect sizes was found between studies (*Q* = 21.98, *I*^*2*^ = 73%, *P* = 0.001) (Fig. 4). A sensitivity test revealed a consistent finding when excluding a cohort study [40]. The funnel plot, Egger’s regression (*b* = -1.82, *p* = 0.21), and Fail-safe-N analyses indicated a non-significant publication bias of included studies (Additional file 4: Appendix 4).

For the association between education level and preventive behaviors, there was not enough data for the meta-analysis. Narratively, four studies indicated mixed results, particularly three reporting no significant difference in combined preventive behaviors [14, 40, 41], while one finding a positive association of education levels with both hand hygiene and facemask wearing [42]. In addition, two studies examined the contribution of ethnicity (white vs. non-white), indicating a significant negative association of white ethnicity with physical distancing, but the association was not statistically significant with disinfecting behaviors [36, 37].

### Psychological correlates of preventive behaviors in children and adolescents

A series of psychological factors were measured in the included studies, such as knowledge, attitude (perceived benefits, barriers), risk perception (perceived susceptibility, severity), social interaction (trust, social norms, moral factors), and psychological well-being (depression, anxiety). Four studies measured behavior-related knowledge [13, 38, 41, 43], among which only two studies examined the association of knowledge with preventive behaviors, indicating a significant positive relationship [13, 38]. In addition, several studies examined the association of diverse psychological factors, e.g. social trust [36, 40], moral disengagement [39, 40], social norms [36, 37, 40], and psychological well-being [37, 41], with preventive behaviors in response to the pandemic, revealing mixed results.

**Fig. 5:**
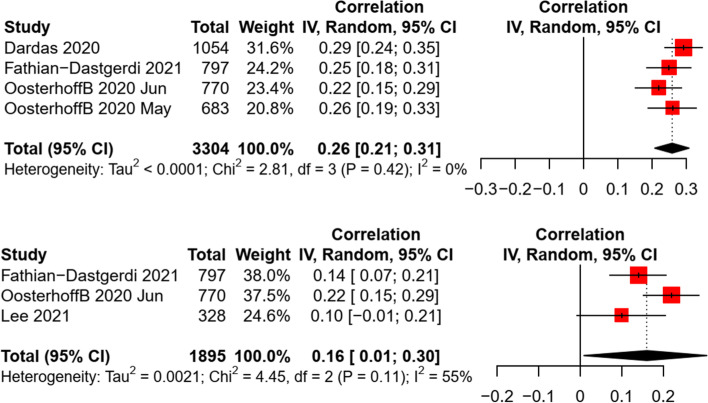
Random effect of attitude and perceived severity on preventive behaviors in children and adolescents

Due to the limited data, the meta-analysis was conducted only for the association of preventive practice with attitude and risk perception (i.e., perceived severity). A significant association between attitude and preventive behaviors was revealed, with a small-to-medium effect size (*k* = 4, *n* = 3,304, *r* = 0.26, *p* < 0.001). Similarly, a small and significant association was found between perceived severity and preventive behaviors in children and adolescents (*k* = 3, *n* = 1,895, *r* = 0.16, *p* = 0.045). The heterogeneity of effect sizes was non-significant between studies (see Fig. 5). A non-significant risk of publication bias was identified by the funnel plots, Egger’s tests (all *p* < 0.05), and Fail-safe-N analyses (see Additional file 4: Appendix 4).

### Social and environmental correlates of preventive behaviors in children and adolescents

**Fig. 6:**
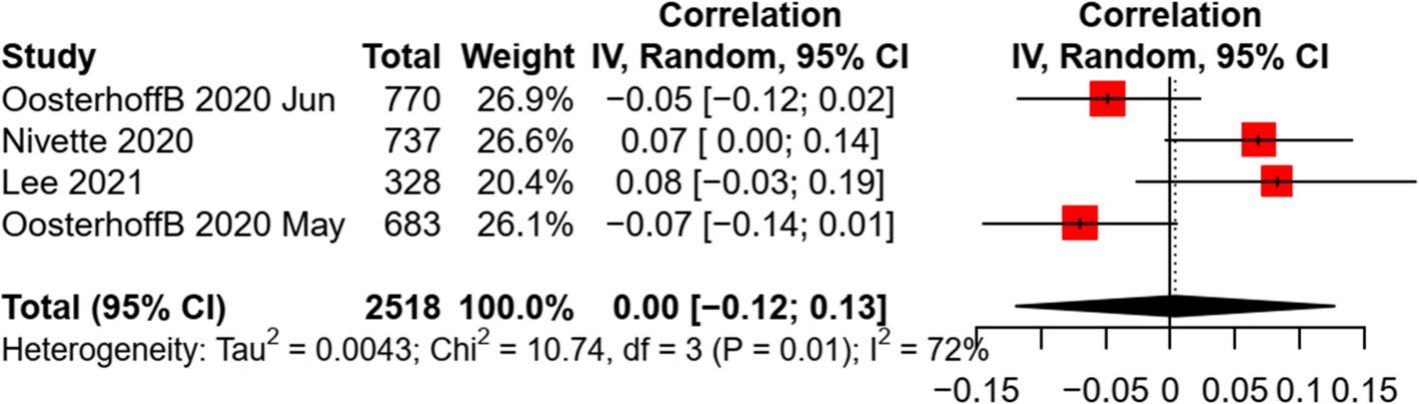
Random effect of family economic status on preventive behaviors in children and adolescents

Several studies examined the association of children and adolescents’ preventive practice with social and environmental factors, such as family residence (e.g., rural/urban), family economic status, parental education levels, and community/city sanitation levels. For the association between residence and preventive behaviors, inconsistent results were found in three studies [13, 38, 41]. Mixed findings were also revealed for the relationship between parental education levels and preventive behaviors in four studies [36–38, 42]. The community/city sanitation levels (e.g., lockdown) was found to have a significant positive association with physical distancing [37], facemask wearing [42], and combined preventive behaviors [17], but negatively associated with hand hygiene [42]. For family economic status, a pooled analysis revealed a non-significant association with preventive behaviors in children and adolescents (*k* = 4, *n* = 2,518, *r* = 0.004, *p* = 0.93). A moderate heterogeneity was found between studies (see Fig. 6). Sensitivity test revealed a consistent finding when excluding a cohort study [40]. The funnel plots, Egger’s tests (*b* = 4.90, *p* = 0.53), and Fail-safe-N analyses indicated a non-significant publication bias (Additional file 4: Appendix 4).

## Discussion

The purpose of this paper was to synthesize and review the evidence for children and adolescent’s practice of COVID-19 preventive behaviors in comparison to adult samples. A secondary purpose was to review current evidence for the demographic, psychological, and social and environmental correlates of the preventive practice among children and adolescents during the pandemic (Table 2). The results are the first quantitative summary statistics for this age group and give new insights into designing effective interventions and making relevant policy-decisions to promote children and adolescents’ preventive behaviors in the fight against the COVID-19 and future pandemics.

**Table 2:** Summary of the findings of this study

Category	Constructs
Demographic factors	Age (ns), gender (girls > boys), education level (mixed)
Psychological factors	Knowledge (narratively positive), attitude (sig +), risk perception (sig +), social interaction (mixed), psychological well-being (mixed)
Social and environmental factors	Family residence (mixed), family economic status (ns), parental education level (mixed), community/city sanitation levels (narratively positive with PD, FW, and combined behaviors; negative with HH)

*Note.* *ns* non-significant association in the meta-analysis, *sig + *significant positive association in the meta-analysis, *PD* physical distancing, *FW* facemask wearing, *HH* hand hygiene

Collectively, this review represents 23 studies with over 25,000 children and adolescents from diverse regions. The majority of studies included in the quantitative analysis were rated as good and high quality (16/17, 94.1%) and there was no statistically significant publication bias for all included studies. The majority of the included studies (18/23, 78.3%) measured the multiple preventive practice combining not only the three major preventive behaviors (i.e., hand hygiene, facemask wearing, physical distancing) but also other preventive measures (e.g., covering coughs, house disinfection, intake vitamin C). In addition to the combination of multiple preventive behaviors, seven studies provided examinations on certain specific behavior, including hand hygiene (n = 4), facemask wearing (n = 2), and physical distancing (n = 5).

For the prevalence of combined preventive behaviors, 16–94% of children and adolescents adopted relevant preventive measures in response to the COVID-19 pandemic. In terms of three major preventive measures, the compliance rate was 76–89%, 60–88%, and 31–87% for hand hygiene, facemask wearing, and physical distancing, respectively. When comparing the preventive practice across different age groups, we found a poorer preventive practice of children and adolescents compared with that of younger adults, while there were non-significant behavioral differences relative to the older adults. This highlights an urgent need of relevant interventions and policies for promoting preventive behaviors in children and adolescents during the pandemic. As the compulsory policy of school closure has been enacted in many countries, the contact pattern and activity of children and adolescents are mainly community and family interactions [44] and personal protection is more expected accordingly. The under expectation of precautionary practice rises the concern that children and adolescents may threaten other susceptible individuals within community and family with virus transmission.

For the demographic correlates of preventive behaviors, a significant association with small effect size was found between gender and preventive behaviors, where girls showed a higher behavioral compliance during the pandemic. Consistent findings were reported in previous studies, which indicated that girls were more inactive and cautious to decrease outdoor activities during other respiratory epidemics [45], while boys had a high risk-taking tendency [46]. Interestingly, previous review studies with adult populations also indicated a higher level of preventive behaviors [45, 47] and utility of preventive care service [48] in females. Thus, there seems to be a robust gendered affect across all ages in terms of the pandemic preventive practice. One potential interpretation might be the personality difference that females tend to be higher in agreeableness [49]. Another explanation might be the difference in the general social position and social roles between males and females (e.g., females are more sanitary in general, who are more likely to be caregivers and thus take precautions more seriously; females spend more time at home and thus more social distance, etc.) [50]. This suggests that more targeted messaging for men and boys is likely needed and further examination on this assumption is needed. In contrast to gender, other demographics such as age, education levels, and ethnicity were not identified as significant correlates of preventive behaviors among children and adolescents, hinting that the same types of intervention approaches may be administered without targeting by these demographic factors. Nevertheless, these findings are concluded narratively due to the limited evidence and more empirical studies on this aspect are warranted.

For psychological correlates of preventive behaviors, only attitudes and perceived severity were identified as consistently significant correlates of COVID-19 preventive behaviors in children and adolescents. As suggested by various psychosocial theories, e.g. TPB and HBM [13, 14], individuals’ health beliefs, cognitive and emotional appraisals for certain behaviors, and risk perception towards the diseases play a crucial role in initiating behaviors. The findings of this study are consistent with previous review articles investigating adult populations [51]. We also found that children and adolescents showed a comparatively lower level of knowledge and attitude than adults. The findings presented here to some extent may provide an explanation as to why children and adolescents demonstrate inferior behavioral compliance during the COVID-19 pandemic. However, it is worth noting that more than half of the included studies did not report the theoretical framework and there was limited data to quantitatively analyze the effect size of other psychosocial correlates (e.g., social norms, intention, self-efficacy). Based on relevant theories and evidence, these factors are important and deserve more research and attention, especially targeting children and adolescents [52]. The above findings particularly suggest the use of psychological theories in future interventions on promoting children and adolescents’ preventive behaviors in fight against the pandemics. For example, “threat” components or information about negative disease consequences could be used sparingly in the interventions. Further, a focus on causal explanation arguments of high positive expectancy benefits and appropriate persuasive peripheral cues (e.g., aiming to form positive attitudes) could be provided [53]. In addition, more research on the maintenance of preventive behaviors is needed by taking several factors other than cognitions (e.g., habit, social identity, self-regulation) into account.

For the family and social correlates of preventive behaviors, mixed results were revealed for a series of factors, such as parental education levels and family residence. It is notable that the community/city sanitation levels were narratively identified as an important correlate of preventive behaviors in children and adolescents and more empirical evidence is needed for quantitative synthesis. In addition, we found a non-significant relationship between family economic status and preventive behaviors among children and adolescents. The findings were inconsistent with previous studies in adult populations. The reason might be that adults determine family economic status rather than the children and adolescents, so the influence of economic status on children and adolescents’ behaviors is relatively weak [54]. This might be also attributed to measurement issues (i.e., implicit measures for family economic status, and diverse types of preventive behaviors).

Despite notable findings in this review, there are several limitations. First, due to the limited data, we were not able to conduct moderator analyses (e.g., cultural contexts, types of preventive behaviors, chronic health condition of children) and future research including testing of potential moderators is warranted. Next, despite our best efforts to implement a thorough literature search of the limited databases, we may have omitted suitable studies by not including key terms over the time span that was searched. The current review only summarized the evidence during the initial stage of the pandemic and further updates are needed, especially for the key correlates of behavioral maintenance. Moreover, a high degree of heterogeneity, and the small number of included studies could result in cautious interpretation of the synthesized results. As a result, any generalizations of the findings to different cultural contexts should be applied with caution and this point deserves further investigation. As the relevant evidence continues to increase, future research syntheses may be able to detect effects of more demographic, psychological, parental, social, and environmental factors of children’s preventive behaviors. Finally, this paper only targeted the general practice of preventive behaviors during the initial stage the COVID-19 pandemic while the synthesis for specific behavior has not been undertaken due to limited evidence. Further examination on the characteristics and distinction of different preventive behaviors is needed.

## Conclusion

To the best of our knowledge, this was the first review and meta-analysis on the compliance and associated factors of preventive behaviors in children and adolescents during the initial stage of the COVID-19 pandemic. Our findings showed that the compliance with preventive behaviors in children and adolescents was significantly lower than younger adults. In addition, small-to-medium overall effects were identified for the associations of COVID-19 preventive behaviors with gender, attitudes, and perceived severity in children and adolescents. Interventions and relevant policy to promot children and adolescent’s compliance with preventive measures should be a priority in the battle against COVID-19 and future pandemics. More studies are warranted to examine the impacts of diverse demographic, psychosocial, and social environmental correlates of children and adolescents’ preventive behaviors during pandemics.

## Implications and contribution

This study has made a better understanding of the practice of preventive behaviors among children and adolescents and its associated factors which is important in designing interventions and relevant policy changes in the battle against pandemics.

## Supplementary Information

**Additional file 1.** Search strategies.

**Additional file 2.** Summary of included studies with their characteristics.

**Additional file 3.** Study quality.

**Additional file 4.** Funnel plots and Fail-safe-N analysis.

**Additional file 5.** PRISMA 2020 checklist.

**Additional file 6.** R syntax.

## Data Availability

All data generated or analysed during this study are included in this published article and its supplementary information files.
