# Supplementary material for: A systematic review and meta-analysis on the preventive behaviors in response to the COVID-19 pandemic among children and adolescents
Source: BMC Public Health. 2022 Jun 15;22:1201. doi: 10.1186/s12889-022-13585-z (PMC9200376; doi:10.1186/s12889-022-13585-z)
Supplement: Supplementary file 1 — Additional file 1. Search strategies. [file 12889_2022_13585_MOESM1_ESM.docx]

**Appendix 1**

Search Strategies

| Databases | Search strategy |
| --- | --- |
| PsyINFO | ab((Coronavirus OR corona* OR COVID* OR COVID-19 OR SARS-CoV-2 OR pandemic OR epidemic) AND (hand washing OR hand hygiene OR hygiene OR face mask OR mask* OR social distancing OR physical distancing OR behav* OR avoid* OR preventive behavior OR precautionary behavior OR coping behavior OR preventive measure OR protect* OR prevent*) AND (demographic* OR sociodemographic* OR socioeconomic* OR psycho* OR psychosocial* OR social* OR "social cognitive" OR factor* OR assoc* OR determin* OR predict* OR antecedent* OR characteristic* OR correlate* OR indicator* OR theor* OR risk factor)) AND pd (20200101-20201231) [Human] |
| PubMed | #1 Title/Abstract: Coronavirus OR corona* OR COVID* OR COVID-19 OR SARS-CoV-2 OR pandemic OR epidemic  #2 Title/Abstract: hand washing OR hand hygiene OR hygiene OR face mask OR mask* OR social distancing OR physical distancing OR behav* OR avoid* OR preventive behavior OR precautionary behavior OR coping behavior OR preventive measure OR protect* OR prevent*  #3 Title/Abstract: demographic* OR sociodemographic* OR socioeconomic* OR psycho* OR psychosocial* OR social* OR "social cognitive" OR factor* OR assoc* OR determin* OR predict* OR antecedent* OR characteristic* OR correlate* OR indicator* OR theor* OR risk factor  #1 AND #2 AND #3  Filters: from 2020/01/01-2020/12/31 [human] |
| Medline-EBSCOhost | #1 AB: Coronavirus OR corona* OR COVID* OR COVID-19 OR SARS-CoV-2 OR pandemic OR epidemic  #2 AB: hand washing OR hand hygiene OR hygiene OR face mask OR mask* OR social distancing OR physical distancing OR behav* OR avoid* OR preventive behavior OR precautionary behavior OR coping behavior OR preventive measure OR protect* OR prevent*  #3 AB: demographic* OR sociodemographic* OR socioeconomic* OR psycho* OR psychosocial* OR social* OR "social cognitive" OR factor* OR assoc* OR determin* OR predict* OR antecedent* OR characteristic* OR correlate* OR indicator* OR theor* OR risk factor  #1 AND #2 AND #3  Filters: from 2020/01/01-2020/12/31 [human] |
| EMBASE (Medline-Ovid) | #1: (Coronavirus or corona* or COVID* or COVID-19 or SARS-CoV-2 or pandemic or epidemic).mp. [mp=title, abstract, heading word, drug trade name, original title, device manufacturer, drug manufacturer, vdevice trade name, keyword, floating subheading word, candidate term word]  #2 limit #1 to human and embase and yr="2020"  #3 (hand washing or hand hygiene or hygiene or face mask or mask* or social distancing or physical distancing or behav* or avoid* or preventive behavior or precautionary behavior or coping behavior or preventive measure or protect* or prevent*).mp. [mp=title, abstract, heading word, drug trade name, original title, device manufacturer, drug manufacturer, device trade name, keyword, floating subheading word, candidate term word]  #4 limit #3 to human and embase and yr="2020"  #5 (demographic* or sociodemographic* or socioeconomic* or psycho* or psychosocial* or social* or "social cognitive" or factor* or assoc* or determin* or predict* or antecedent* or characteristic* or correlate* or indicator* or theor* or risk factor).mp. [mp=title, abstract, heading word, drug trade name, original title, device manufacturer, drug manufacturer, device trade name, keyword, floating subheading word, candidate term word]  #6 limit #5 to to human and yr="2020"  #2 AND #4 AND #6 |
| Cochrane Library | #1 Title/Abstract/keywords: Coronavirus OR corona* OR COVID* OR COVID-19 OR SARS-CoV-2 OR pandemic OR epidemic  #2 Title/Abstract: hand washing OR hand hygiene OR hygiene OR face mask OR mask* OR social distancing OR physical distancing OR behav* OR avoid* OR preventive behavior OR precautionary behavior OR coping behavior OR preventive measure OR protect* OR prevent*  #3 Title/Abstract: demographic* OR sociodemographic* OR socioeconomic* OR psycho* OR psychosocial* OR social* OR "social cognitive" OR factor* OR assoc* OR determin* OR predict* OR antecedent* OR characteristic* OR correlate* OR indicator* OR theor* OR risk factor  #1 AND #2 AND #3  Filters: from 2020/01/01-2020/12/31 |
