# Supplementary material for: A systematic review and meta-analysis on the preventive behaviors in response to the COVID-19 pandemic among children and adolescents
Source: BMC Public Health. 2022 Jun 15;22:1201. doi: 10.1186/s12889-022-13585-z (PMC9200376; doi:10.1186/s12889-022-13585-z)
Supplement: Supplementary file 2 — Additional file 2. Summary of included studies with their characteristics. [file 12889_2022_13585_MOESM2_ESM.docx]

| **Appendix 2: Summary of included studies with their characteristics** | | | | | | | | | | |
| --- | --- | --- | --- | --- | --- | --- | --- | --- | --- | --- |
| **Author** | **Region** | **Study design** | **Data collection period** | **Sample size^a^** | **Age (y)** | **Gender of children and adolescents** | **PB measured** | | **Practice of PB^b^** | **Correlates of PB measured** |
| **Compliance with preventive behavior to COVID-19 pandemic among children and adolescents compared with adults** | | | | | | | | | | |
| Shahabi  (Dec 2020) * | Iran | Cross-sectional | Mar-Apr | 161  (1,863) | 15-20 | CD | MPB (Practice of KAP) | | 34.3±6.6 (40) | Less practice, knowledge, and attitude than adults (> 20 y) |
| Alhazmi  (Sep 2020) | Saudi Arabia | Cross-sectional | Apr 24-May 27 | 51  (1,462) | < 18 | CD | MPB (Practice of KAP) | | 76.8±16.2 (100) | Less practice and knowledge, but comparable attitude than adults (≥ 18 y) |
| Honarvar  (Jun 2020) * | Iran | Cross-sectional^2^ | Feb-Mar | 133 (1,198) | 15-19 | CD | MPB (Practice of KAP) | | 51.7±9.3 (70) | Less practice and knowledge than adults ≥ 20 y) |
| Ferdous  (Oct 2020) * | Bangladesh | Cross-sectional | Mar 29- Apr 20 | 671  (1,346) | 12-20 | CD | MPB (Practice of KAP) | | 90.8-93.8% | Less practice and attitude, but more knowledge than adults (> 30 y) |
| Narayana  (Jul 2020) | India | Cross-sectional | May 1-15 | 33  (2,426) | <18 | CD | MPB (PD, HH, FMW) | | 7.6±1.8 (10) | Less practice and knowledge than adults ≥ 26 y) |
| Cui  (Nov 2020) * | China | Cross-sectional | Feb 15-Apr 21 | 5,342  (46,724) | < 20 | CD | MPB (PD, HH, FMW) | | 31.2±3.4 (33) | Less practice and knowledge than adults (21-60 y) except elderly (≥ 61 y) |
| Ye  (Sep 2020) * | China | Cross-sectional^2^ | Apr 04- Apr 15 | 599  (4,189) | 10-19 | CD | MPB (HH, FMW) | | 53.1% | Less MPB than adults (≥ 21 y) except middle aged (41-60 y) |
| Wang  (Oct 2020) | China | Cross-sectional | Mar 1-16 | 774  (4,265) | < 20 | CD | PD; HH; FMW | | 86.7; 75.7; 87.7% | Less FMW than adults (31-50 y), less HH than adults (31-40 y), and less risk perception than adults (≥ 31 y) |
| Desalegn  (Jan 2021) * | Ethiopia | Cross-sectional | Mar 24-Apri 7 | 48  (791) | 18-19 | CD | MPB (Practice of KAP) | | 46.1-60.1% | Comparable practice and attitude, but less knowledge than adults (> 20 y) |
| Pinchoff  (Dec 2020) | India | Longitudinal | Dataset 2015/16- 2018/19; 2020 NR | 248  (1418) | 18-19 | Girls: 64.5%; Boys: 35.5% | MPB (PD, HH, FMW) | | 21% | No significant difference in knowledge and practice compared to young adults (20-24 y) |
| Firouzbakht  (Jan 2021) * | Iran | Cross-sectional | Mar 25- Apr 5 | 83  (2,097) | 16-20 | Girls: 73.5%; Boys: 26.5% | MPB (PB, HH) | | 71.7±2.5% | Comparable preventive behavior than adults (> 20 y); gender, education level, family income, health condition |
| **Correlates of preventive behaviors among children and adolescents** | | | | | | | | | | |
|  |  |  |  |  |  |  |  | | |  |
| Dardas  (Sep 2020) * | Jordan | Cross-sectional^2^ | NR | 1,054 | 12-18 | Girls: 71.0%; Boys: 29.0% | MPB (Practice of KAP) | 23 (28) | | Age, gender, residence, knowledge, attitude |
| Xue  (Jan 2021) * | China | Cross-sectional | Feb-Mar | 1,650 | CD | Girls: 43.4%; Boys: 56.6% | MPB (Practice of KAP) | 8.7±1.7 (10) | | Gender, knowledge, residence, grade, depression, anxiety |
| Guzek  (Aug 2020) | Poland | Cross-sectional | Mar 31- Apr 29 | 2,323 | 15-20 | Girls: 65.0%; Boys: 35.0% | MPB (PD, HH, FMW) | NC | | Gender, knowledge |
| Ogubuike  (Jan 2021) | Nigeria | Cross-sectional | Jul-Aug | 200 | 11-16 | Girls: 52.0%; Boys: 48.0% | MPB (PD, HH, FMW) | 16-58% | | Age, gender, parents staying together |
|  |  |  |  |  |  |  |  |  | |  |
| Fathian-Dastgerdi  (Jan 2021) * | Iran | Cross-sectional^2^ | May 28- Jun 28 | 797 | 12-18 | Girls: 53.7%; Boys: 46.3% | MPB (PD, HH, FMW) | NR | | Gender, grade, family income, perceived susceptibility, perceived severity, attitude (perceived benefits, perceived barrier), self-efficacy |
| OosterhoffB  (Jun 2020) | US | Cross-sectional | Mar 20 – Mar 22 | 770 | 13-18 | Girls: 74.7%; Boys: 20.3%;  Other: 5.1% | MPB (PD, HH); PD | 87.8; 31.4% | | Age, gender, race, Hispanic, financial strain, parents’ education level, ideology, severity, social responsibility, social trust, self-interest, news monitoring |
| Nivette  (Sep 2020) * | Switzerland | Longitudinal^4^ | Dataset 2004-2018; Mar-Apr 2020 | 737 | 17-20 | Girls: 49.0%; Boys: 51.0% | MPB (PD, HH); PD; HH | 82-97; 88; 73% | | Gender, socioeconomics, education, migrant background, social bonds and lifestyle, attitudes toward the law and police, deviant peers and behavior, dispositional factors, self-efficacy, social norm and moral disengagement, trust in government, information-seeking |
| Lee  (Jan 2021) * | South Korea | Cross-sectional^3^ | Sep-Oct | 328 | 12-16 | Girls: 44.5%; Boys: 55.5% | MPB (PD) | 16.9±2.8% | | Age, gender, health status, family factors (family income, family satisfaction), environmental factors (levels of sanitation since COVID-19, perceptions regarding the risk of COVID-19) |
| Alivernini  (Feb 2020) * | Italy | Longitudinal^1^ | 2019; 2020 May- Jun | 347 | 14-19 | Girls: 42.1%; Boys: 57.9% | PD | NR | | Age, gender, motivation, moral disengagement, intention, personality traits |
| OosterhoffB  (May 2020) * | US | Cross-sectional^1^ | Mar 29- Mar 30 | 683 | 13-18 | Girls: 75.3%; Boys: 22.7%;  Other: 4.9% | PD | 16.5-83.5% | | Age, gender, race, Hispanic, financial strain, parents’ education, lockdown, parents’ rules, peers’ perception, social responsibility, avoid personal sick, avoid other sick, no alternatives, prefer to stay home, avoid judgment, anxiety, depression, burdensomeness, belongingness |
| Riiser  (Aug 2020) | Norway | Cross-sectional | Mar-Apr | 2,205 | 16-19 | Girls: 82.5%; Boys: 17.2% | PD; HH | 86.4; 66.7-88.9% | | Age, gender, corona infection, living situation, residence, parents’ education level, parents’ country of birth, actual knowledge (health literacy) |
| Chen  (Apr 2020) * | China | Cross-sectional | Feb 16-25 | 8,569 | 6-13 | Girls: 47.3%; Boys: 52.7% | HH; FMW | 40.8-43.5; 59.2% | | Gender, grade, out-going history, parents’ educational background and occupation, residence, lock-down |

Note. * Studies included in the meta-analysis; CD = cannot decide (although these studies included a subgroup of children and adolescents, there was no relevant analysis for the subgroup); NA, not applicable; NR, KAP = Knowledge, Attitude and Practice; PB = preventive behaviors; MPB, multiple preventive behaviors; ^a^ data expressed as no. of target group (no. of adults for comparison) or no. of target group; ^b^ mean score (total score) or percentage of compliance with PB, NC, not clear, NR, not report; ^1-5^ Theoretical backdrop reported: ^1^= Self-determination Theory, ^2^ = Health Belief Model, ^3^ = Ecological System, ^4^ = Integrated Cognitive Antisocial Potential (ICAP) .
