# Supplementary material for: A systematic review and meta-analysis on the preventive behaviors in response to the COVID-19 pandemic among children and adolescents
Source: BMC Public Health. 2022 Jun 15;22:1201. doi: 10.1186/s12889-022-13585-z (PMC9200376; doi:10.1186/s12889-022-13585-z)
Supplement: Supplementary file 3 — Additional file 3. Study quality. [file 12889_2022_13585_MOESM3_ESM.docx]

| **Article** | **#1** | **#2** | **#3** | **#4** | **#5** | **#6** | **#7** | **#8** | **#9** | **#10** | **#11** | **#12** | **#13** | **#14** | **Quality** |
| --- | --- | --- | --- | --- | --- | --- | --- | --- | --- | --- | --- | --- | --- | --- | --- |
| Alhazmi (Sep 2020) | **Y** | **Y** | **Y** | **Y** | **N** | **Y** | **NA** | **Y** | **Y** | **NA** | **NR** | **NA** | **NA** | **Y** | Moderate |
| Alivernini (Feb 2020) | **Y** | **Y** | **Y** | **Y** | **N** | **Y** | **NA** | **Y** | **Y** | **NA** | **Y** | **NA** | **NA** | **Y** | Good |
| Chen (Apr 2020) | **Y** | **Y** | **Y** | **Y** | **N** | **Y** | **NA** | **Y** | **Y** | **NA** | **Y** | **NA** | **NA** | **Y** | Good |
| Cui (Nov 2020) | **Y** | **Y** | **Y** | **Y** | **N** | **Y** | **NA** | **Y** | **Y** | **NA** | **Y** | **NA** | **NA** | **Y** | Good |
| Dardas (Sep 2020) | **Y** | **Y** | **Y** | **Y** | **Y** | **Y** | **NA** | **Y** | **Y** | **NA** | **Y** | **NA** | **NA** | **Y** | High |
| Desalegn (Jan 2021) | **Y** | **Y** | **Y** | **Y** | **Y** | **Y** | **NA** | **Y** | **Y** | **NA** | **Y** | **NA** | **NA** | **Y** | High |
| Fathian-Dastgerdi (Jan 2021) | **Y** | **Y** | **Y** | **Y** | **Y** | **Y** | **NA** | **Y** | **Y** | **NA** | **Y** | **NA** | **NA** | **Y** | High |
| Ferdous (Oct 2020) | **Y** | **Y** | **Y** | **NR** | **N** | **Y** | **NA** | **Y** | **Y** | **NA** | **Y** | **NA** | **NA** | **Y** | Moderate |
| Firouzbakht (Jan 2021) | **Y** | **Y** | **Y** | **Y** | **N** | **Y** | **NA** | **Y** | **Y** | **NA** | **Y** | **NA** | **NA** | **Y** | Good |
| Guzek (Aug 2020) | **Y** | **Y** | **Y** | **Y** | **N** | **Y** | **NA** | **Y** | **Y** | **NA** | **Y** | **NA** | **NA** | **NA** | Good |
| Honarvar (Jun 2020) | **Y** | **Y** | **Y** | **Y** | **Y** | **Y** | **NA** | **Y** | **Y** | **NA** | **Y** | **NA** | **NA** | **Y** | High |
| Lee (Jan 2021) | **Y** | **Y** | **Y** | **Y** | **N** | **Y** | **NA** | **Y** | **Y** | **NA** | **Y** | **NA** | **NA** | **Y** | Good |
| Narayana (Jul 2020) | **Y** | **Y** | **Y** | **Y** | **Y** | **Y** | **NA** | **Y** | **Y** | **NA** | **Y** | **NA** | **NA** | **Y** | High |
| Nivette (Sep 2020) | **Y** | **Y** | **Y** | **Y** | **N** | **Y** | **NA** | **Y** | **Y** | **NA** | **Y** | **NA** | **NA** | **Y** | High |
| Ogubuike (Jan 2021) | **Y** | **Y** | **Y** | **Y** | **N** | **Y** | **NA** | **Y** | **Y** | **NA** | **N** | **NA** | **NA** | **Y** | Moderate |
| OosterhoffB (May 2020) | **Y** | **Y** | **Y** | **NR** | **Y** | **Y** | **NA** | **Y** | **Y** | **NA** | **Y** | **NA** | **NA** | **Y** | Good |
| OosterhoffB (Jun 2020) | **Y** | **Y** | **Y** | **Y** | **N** | **Y** | **NA** | **Y** | **Y** | **NA** | **Y** | **NA** | **NA** | **Y** | Good |
| Pinchoff (Dec 2020) | **Y** | **Y** | **Y** | **NR** | **N** | **Y** | **NA** | **Y** | **Y** | **NA** | **Y** | **NA** | **NA** | **Y** | Moderate |
| Riiser (Aug 2020) | **Y** | **Y** | **Y** | **Y** | **N** | **Y** | **NA** | **Y** | **Y** | **NA** | **Y** | **NA** | **NA** | **Y** | Good |
| Shahabi (Dec 2020) | **Y** | **Y** | **Y** | **Y** | **N** | **Y** | **NA** | **Y** | **Y** | **NA** | **Y** | **NA** | **NA** | **Y** | Good |
| Wang (Oct 2020) | **Y** | **Y** | **Y** | **Y** | **Y** | **Y** | **NA** | **Y** | **Y** | **NA** | **Y** | **NA** | **NA** | **Y** | High |
| Xue (Jan 2021) | **Y** | **Y** | **Y** | **Y** | **N** | **Y** | **NA** | **Y** | **Y** | **NA** | **Y** | **NA** | **NA** | **Y** | Good |
| Ye (Sep 2020) | **Y** | **Y** | **Y** | **Y** | **N** | **Y** | **NA** | **Y** | **Y** | **NA** | **Y** | **NA** | **NA** | **Y** | Good |

Note. #1: research question or objective; #2: study population; #3: participation rate; #4: inclusion criteria; #5: sample size estimation; #6: exposure interest measured; #7: timeframe of measurement; #8: examination of different levels of the exposure; #9: clearly defined exposure measures; #10: exposure assessed more than once over time; #11: clear definition, reliability and validity of outcome measures; #12: outcome assessors blinded; #13: dropout rate; #14: cofounding variables measures and adjusted; Y = Yes (i.e., clearly and appropriately addressed); N = No; NA = Not Applicable; NR = Not Reported.
