# Supplementary material for: A systematic review and meta-analysis on the preventive behaviors in response to the COVID-19 pandemic among children and adolescents
Source: BMC Public Health. 2022 Jun 15;22:1201. doi: 10.1186/s12889-022-13585-z (PMC9200376; doi:10.1186/s12889-022-13585-z)
Supplement: Supplementary file 4 — Additional file 4. Funnel plots and Fail-safe-N analysis. [file 12889_2022_13585_MOESM4_ESM.pdf]

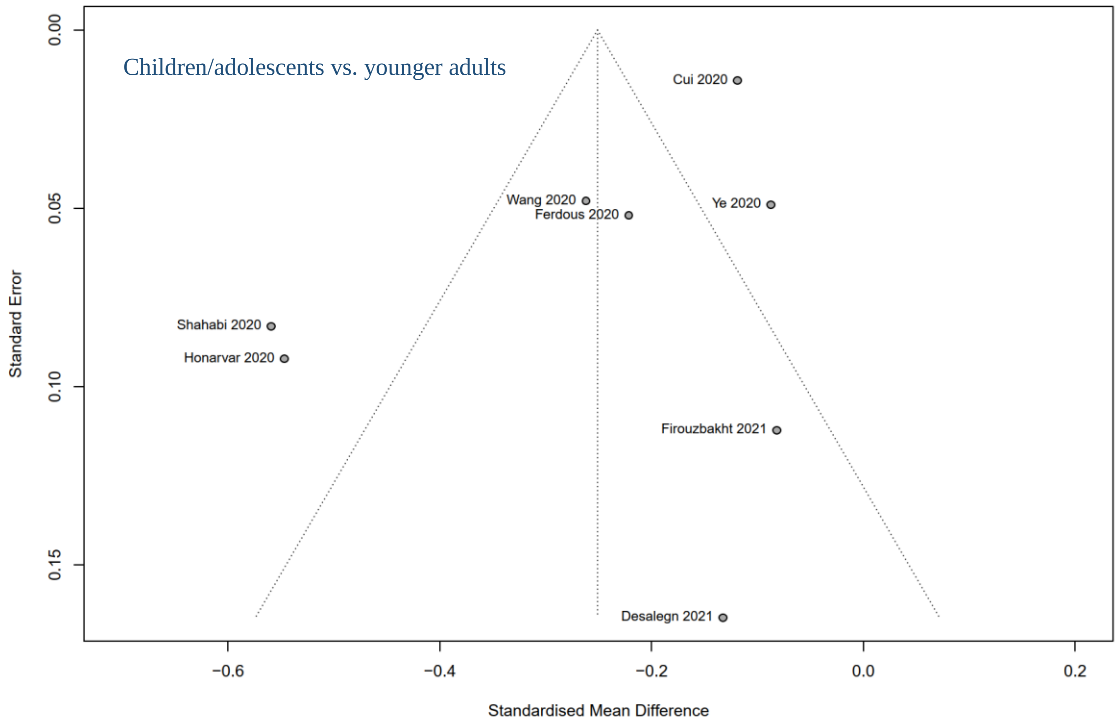
Children/adolescents vs. younger adults
Cui 2020
Wang 2020
Ferdous 2020
Ye 2020
Shahabi 2020
Honarvar 2020
Firouzbakht 2021
Desalegn 2021
Standard Error
0.00
0.05
0.10
0.15
−0.6
−0.4
−0.2
0.0
0.2
Standardised Mean Difference

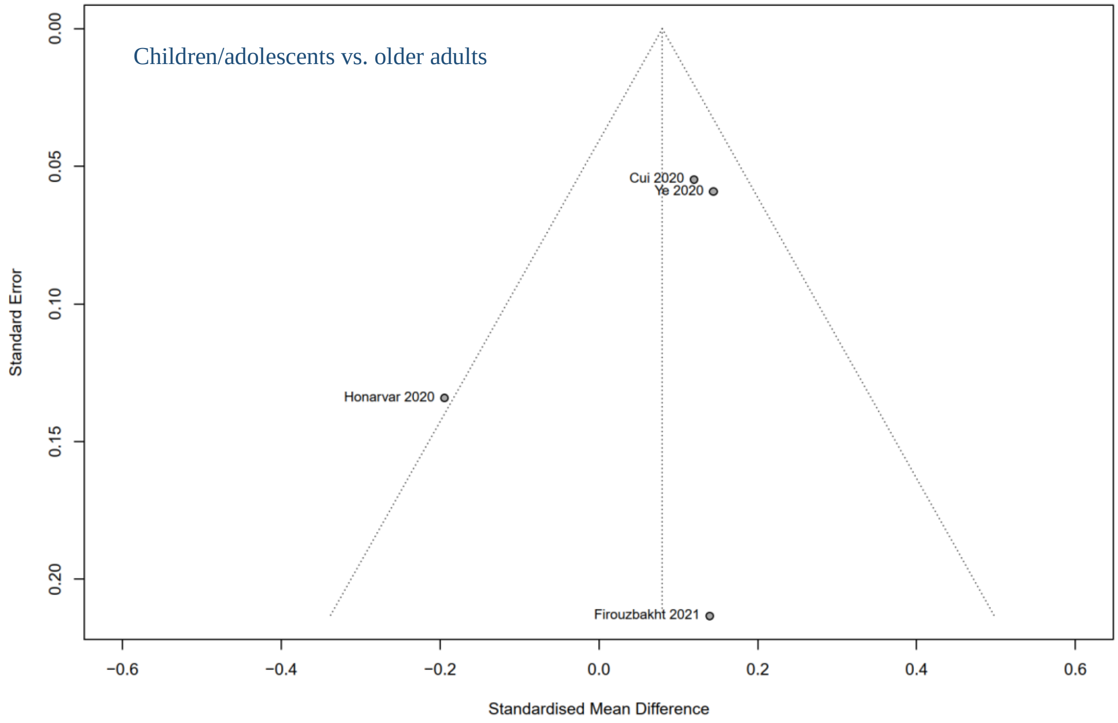
Children/adolescents vs. older adults
Cui 2020
Ye 2020
Honarvar 2020
Firouzbakht 2021
Standard Error
0.00
0.05
0.10
0.15
0.20
−0.6
−0.4
−0.2
0.0
0.2
0.4
0.6
Standardised Mean Difference

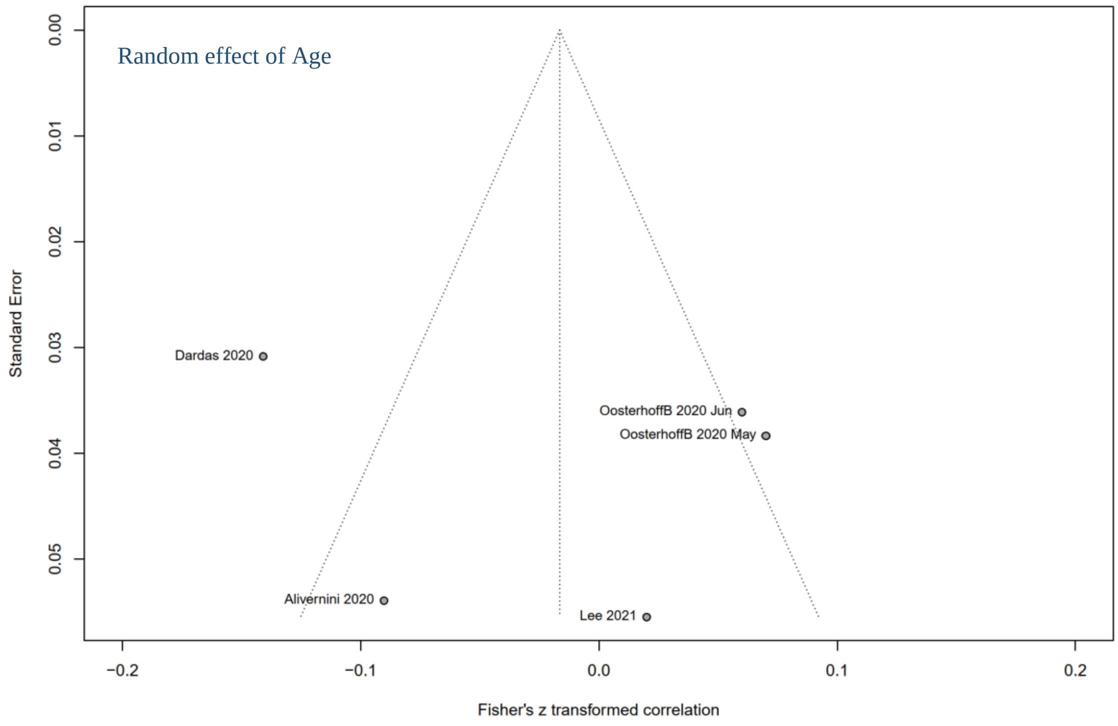
Random effect of Age
Standard Error
0.00
0.01
0.02
0.03
0.04
0.05
Dardas 2020
OosterhoffB 2020 Jun
OosterhoffB 2020 May
Alivernini 2020
Lee 2021
−0.2
−0.1
0.0
0.1
0.2
Fisher's z transformed correlation

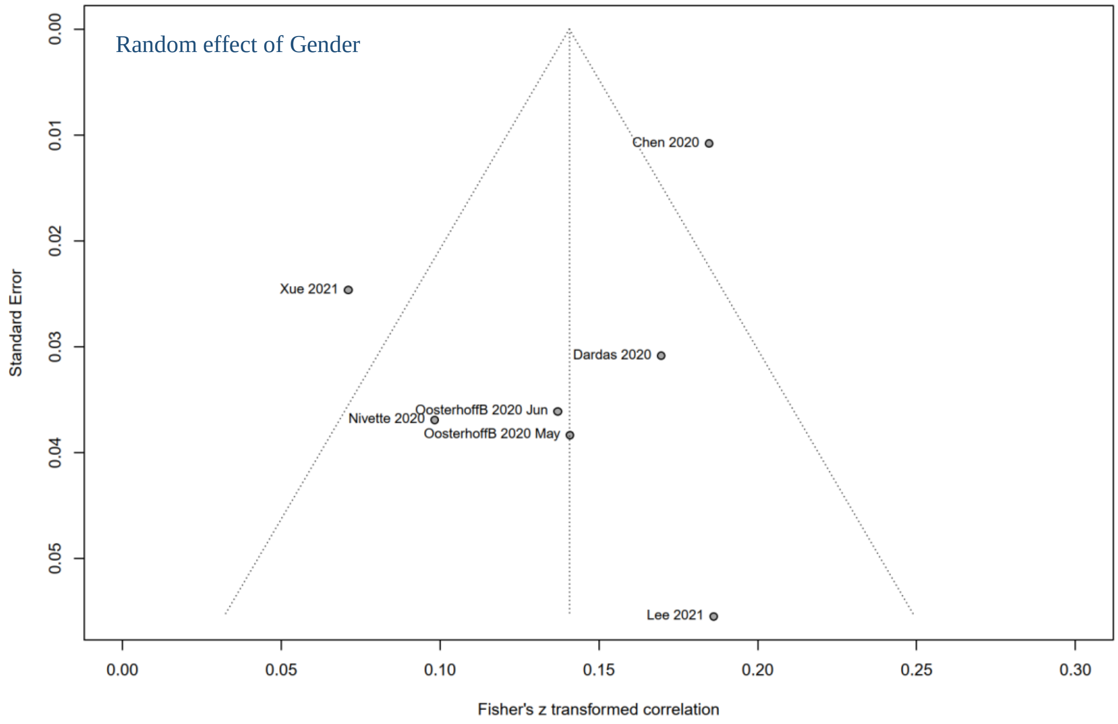
Random effect of Gender
Standard Error
0.00
0.01
0.02
0.03
0.04
0.05
Chen 2020
Xue 2021
Dardas 2020
OosterhoffB 2020 Jun
Nivette 2020
OosterhoffB 2020 May
Lee 2021
0.00
0.05
0.10
0.15
0.20
0.25
0.30
Fisher's z transformed correlation

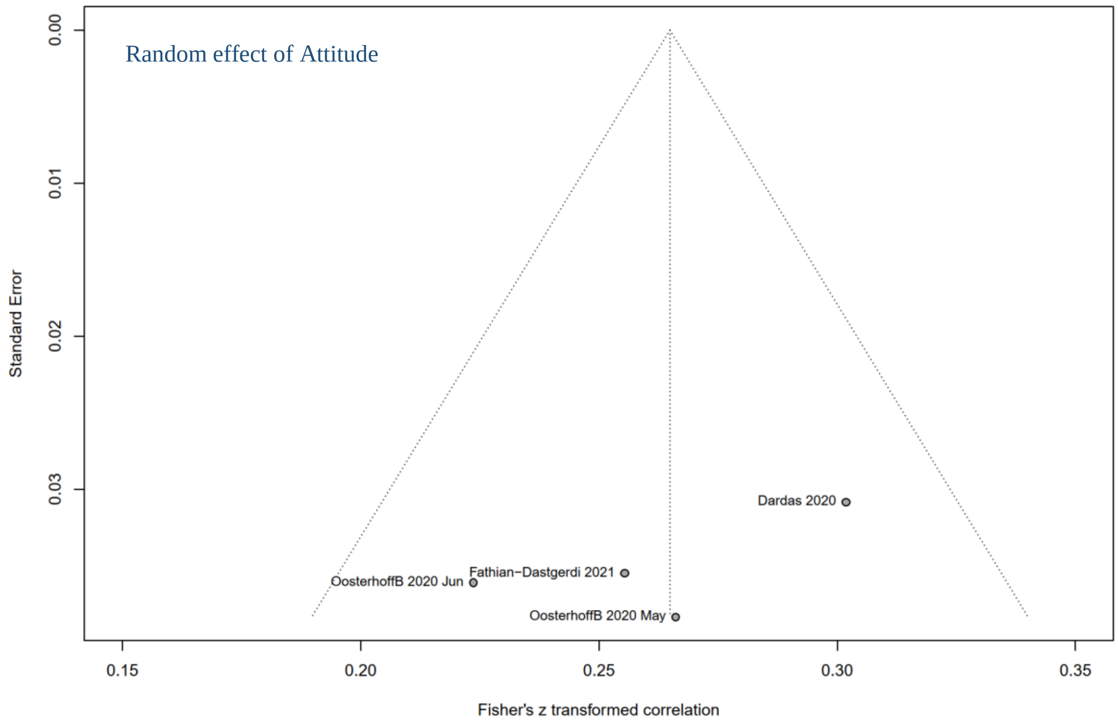

Random effect of Attitude
Standard Error
0.00
0.01
0.02
0.03
Dardas 2020
Fathian−Dastgerdi 2021
OosterhoffB 2020 Jun
OosterhoffB 2020 May
0.15
0.20
0.25
0.30
0.35
Fisher's z transformed correlation

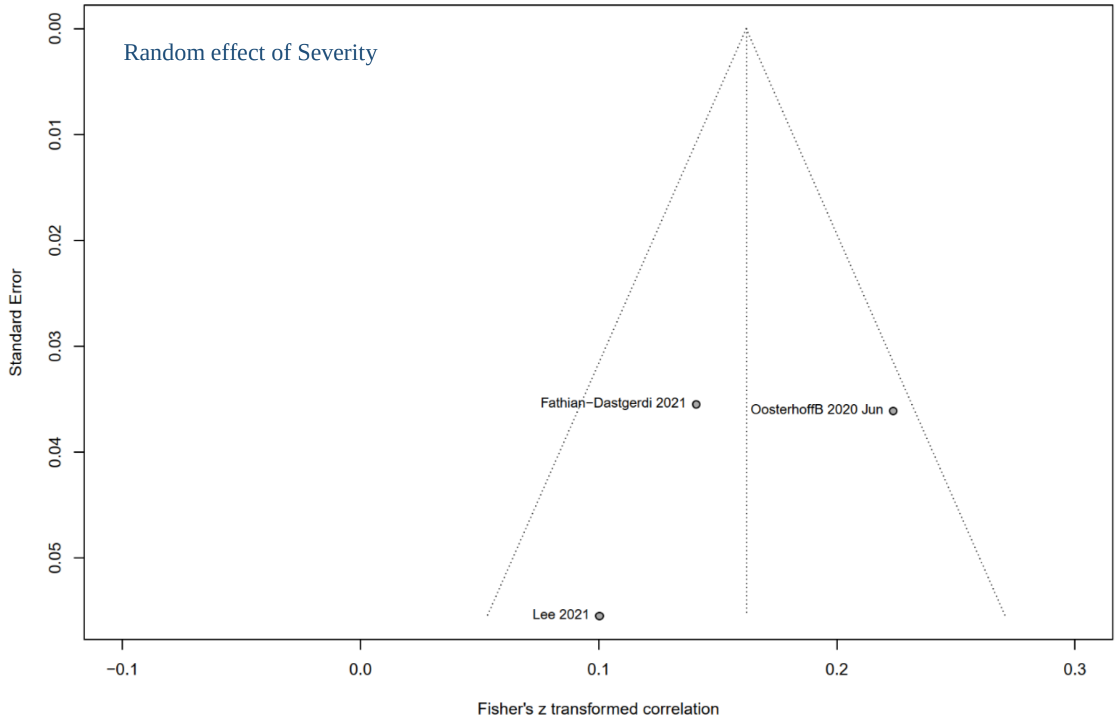

Random effect of Severity
Standard Error
0.00
0.01
0.02
0.03
0.04
0.05
Fathian−Dastgerdi 2021
OosterhoffB 2020 Jun
Lee 2021
−0.1
0.0
0.1
0.2
0.3
Fisher's z transformed correlation

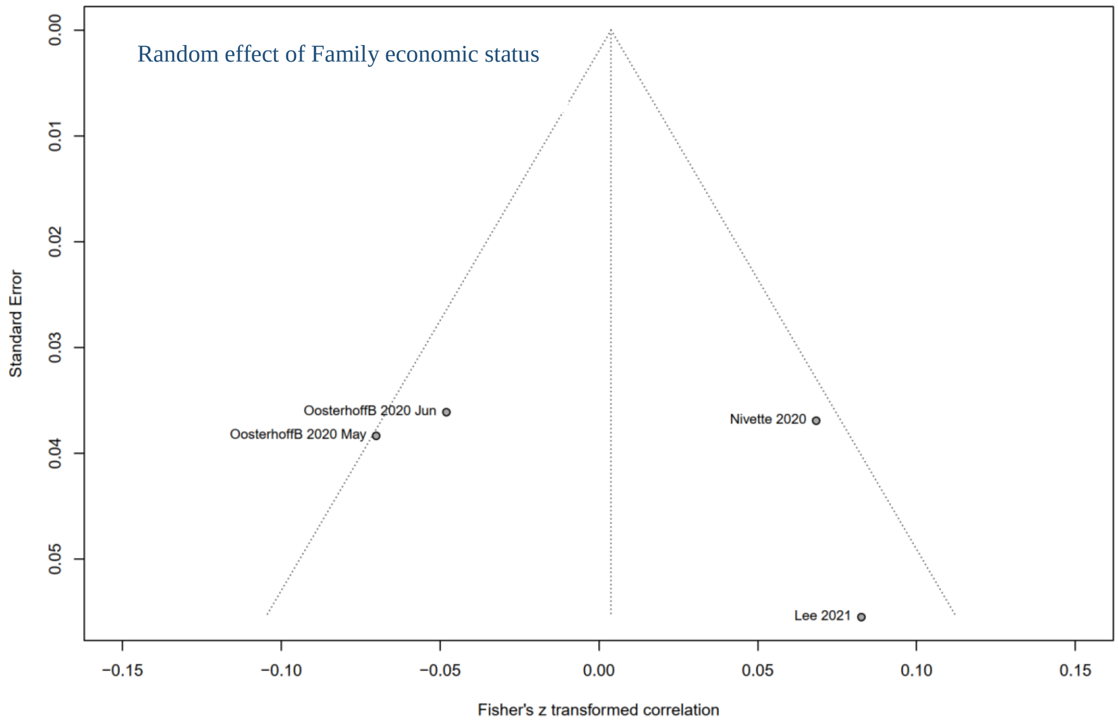
Random effect of Family economic status
Standard Error
0.00
0.01
0.02
0.03
0.04
0.05
OosterhoffB 2020 Jun
OosterhoffB 2020 May
Nivette 2020
Lee 2021
−0.15
−0.10
−0.05
0.00
0.05
0.10
0.15
Fisher's z transformed correlation

Fail-safe-N analyses:

Children vs adult
Fail-safe N Calculation Using the Rosenthal Approach
Observed Significance Level: 0.0015
Target Significance Level: 0.05
Fail-safe N: 19
Average treatment effect [95% prediction interval]:
-0.2233 [-0.5686, 0.0900]
df.: 7
Average treatment effect [95% confidence interval]:
-0.2233 [-0.3896, -0.0695]
df.: 7

Children vs. older adult
Fail-safe N Calculation Using the Rosenthal Approach
Observed Significance Level: 0.3313
Target Significance Level: 0.05
Fail-safe N: 0
Average treatment effect [95% prediction interval]:
0.0836 [-0.3917, 0.5009]
df.: 3
Average treatment effect [95% confidence interval]:
0.0836 [-0.1535, 0.2930]
df.: 3

Age:
Fail-safe N Calculation Using the Rosenthal Approach
Observed Significance Level: 0.1426
Target Significance Level: 0.05
Fail-safe N: 0
Average treatment effect [95% prediction interval]:
-0.0162 [-0.4042, 0.3742]
df.: 4
Average treatment effect [95% confidence interval]:
-0.0162 [-0.1365, 0.1007]
df.: 4

Gender:
Fail-safe N Calculation Using the Rosenthal Approach
Observed Significance Level: <.0001
Target Significance Level: 0.05
Fail-safe N: 555
Average treatment effect [95% prediction interval]:
0.1400 [-0.0494, 0.3275]
df.: 6
Average treatment effect [95% confidence interval]:
0.1400 [0.0997, 0.1796]
df.: 6

Attitude:
Fail-safe N Calculation Using the Rosenthal Approach
Observed Significance Level: <.0001
Target Significance Level: 0.05
Fail-safe N: 332
Average treatment effect [95% prediction interval]:
0.2618 [0.1369, 0.3840]
df.: 3
Average treatment effect [95% confidence interval]:
0.2618 [0.2094, 0.3133]
df.: 3

Perceived severity
Fail-safe N Calculation Using the Rosenthal Approach
Observed Significance Level: <.0001
Target Significance Level: 0.05
Fail-safe N: 50
Average treatment effect [95% prediction interval]:
0.1550 [-0.1909, 0.5071]
df.: 2
Average treatment effect [95% confidence interval]:
0.1550 [-0.0090, 0.3123]
df.: 2
