# Supplementary material for: A systematic review and meta-analysis on the preventive behaviors in response to the COVID-19 pandemic among children and adolescents
Source: BMC Public Health. 2022 Jun 15;22:1201. doi: 10.1186/s12889-022-13585-z (PMC9200376; doi:10.1186/s12889-022-13585-z)
Supplement: Supplementary file 5 — Additional file 5. PRISMA 2020 checklist. [file 12889_2022_13585_MOESM5_ESM.pdf]

PRISMA 2020 Checklist

Section and Topic	Item #	Checklist item	Location where item is reported
**TITLE**			
Title	1	Identify the report as a systematic review.	L1
**ABSTRACT**			
Abstract	2	See the PRISMA 2020 for Abstracts checklist.	L37
**INTRODUCTION**			
Rationale	3	Describe the rationale for the review in the context of existing knowledge.	L71
Objectives	4	Provide an explicit statement of the objective(s) or question(s) the review addresses.	L135
**METHODS**			
Eligibility criteria	5	Specify the inclusion and exclusion criteria for the review and how studies were grouped for the syntheses.	L147
Information sources	6	Specify all databases, registers, websites, organisations, reference lists and other sources searched or consulted to identify studies. Specify the date when each source was last searched or consulted.	L163
Search strategy	7	Present the full search strategies for all databases, registers and websites, including any filters and limits used.	L147
Selection process	8	Specify the methods used to decide whether a study met the inclusion criteria of the review, including how many reviewers screened each record and each report retrieved, whether they worked independently, and if applicable, details of automation tools used in the process.	L147
Data collection process	9	Specify the methods used to collect data from reports, including how many reviewers collected data from each report, whether they worked independently, any processes for obtaining or confirming data from study investigators, and if applicable, details of automation tools used in the process.	L188
Data items	10a	List and define all outcomes for which data were sought. Specify whether all results that were compatible with each outcome domain in each study were sought (e.g. for all measures, time points, analyses), and if not, the methods used to decide which results to collect.	L188
	10b	List and define all other variables for which data were sought (e.g. participant and intervention characteristics, funding sources). Describe any assumptions made about any missing or unclear information.	L188
Study risk of bias assessment	11	Specify the methods used to assess risk of bias in the included studies, including details of the tool(s) used, how many reviewers assessed each study and whether they worked independently, and if applicable, details of automation tools used in the process.	L177
Effect measures	12	Specify for each outcome the effect measure(s) (e.g. risk ratio, mean difference) used in the synthesis or presentation of results.	L206
Synthesis methods	13a	Describe the processes used to decide which studies were eligible for each synthesis (e.g. tabulating the study intervention characteristics and comparing against the planned groups for each synthesis (item #5)).	L206
	13b	Describe any methods required to prepare the data for presentation or synthesis, such as handling of missing summary statistics, or data conversions.	L206
	13c	Describe any methods used to tabulate or visually display results of individual studies and syntheses.	L206
	13d	Describe any methods used to synthesize results and provide a rationale for the choice(s). If meta-analysis was performed, describe the model(s), method(s) to identify the presence and extent of statistical heterogeneity, and software package(s) used.	L216
	13e	Describe any methods used to explore possible causes of heterogeneity among study results (e.g. subgroup analysis, meta-regression).	L226
	13f	Describe any sensitivity analyses conducted to assess robustness of the synthesized results.	L226
Reporting bias assessment	14	Describe any methods used to assess risk of bias due to missing results in a synthesis (arising from reporting biases).	L226
Certainty assessment	15	Describe any methods used to assess certainty (or confidence) in the body of evidence for an outcome.	L226

PRISMA 2020 Checklist

Section and Topic	Item #	Checklist item	Location where item is reported
**RESULTS**			
Study selection	16a	Describe the results of the search and selection process, from the number of records identified in the search to the number of studies included in the review, ideally using a flow diagram.	L234
	16b	Cite studies that might appear to meet the inclusion criteria, but which were excluded, and explain why they were excluded.	L234
Study characteristics	17	Cite each included study and present its characteristics.	Appendix
Risk of bias in studies	18	Present assessments of risk of bias for each included study.	Appendix
Results of individual studies	19	For all outcomes, present, for each study: (a) summary statistics for each group (where appropriate) and (b) an effect estimate and its precision (e.g. confidence/credible interval), ideally using structured tables or plots.	L263
Results of syntheses	20a	For each synthesis, briefly summarise the characteristics and risk of bias among contributing studies.	L263
	20b	Present results of all statistical syntheses conducted. If meta-analysis was done, present for each the summary estimate and its precision (e.g. confidence/credible interval) and measures of statistical heterogeneity. If comparing groups, describe the direction of the effect.	L263
	20c	Present results of all investigations of possible causes of heterogeneity among study results.	L263
	20d	Present results of all sensitivity analyses conducted to assess the robustness of the synthesized results.	L263
Reporting biases	21	Present assessments of risk of bias due to missing results (arising from reporting biases) for each synthesis assessed.	L263
Certainty of evidence	22	Present assessments of certainty (or confidence) in the body of evidence for each outcome assessed.	L263
**DISCUSSION**			
Discussion	23a	Provide a general interpretation of the results in the context of other evidence.	L366
	23b	Discuss any limitations of the evidence included in the review.	L458
	23c	Discuss any limitations of the review processes used.	L458
	23d	Discuss implications of the results for practice, policy, and future research.	L366
**OTHER INFORMATION**			
Registration and protocol	24a	Provide registration information for the review, including register name and registration number, or state that the review was not registered.	L343
	24b	Indicate where the review protocol can be accessed, or state that a protocol was not prepared.	L343
	24c	Describe and explain any amendments to information provided at registration or in the protocol.	NA
Support	25	Describe sources of financial or non-financial support for the review, and the role of the funders or sponsors in the review.	L505
Competing interests	26	Declare any competing interests of review authors.	L499
Availability of data, code and other materials	27	Report which of the following are publicly available and where they can be found: template data collection forms; data extracted from included studies; data used for all analyses; analytic code; any other materials used in the review.	L512

From: Page MJ, McKenzie JE, Bossuyt PM, Boutron I, Hoffmann TC, Mulrow CD, et al. The PRISMA 2020 statement: an updated guideline for reporting systematic reviews. BMJ 2021;372:n71. doi: 10.1136/bmj.n71

MOOSE (Meta-analyses Of Observational Studies in Epidemiology) Checklist

A reporting checklist for Authors, Editors, and Reviewers of Meta-analyses of Observational Studies. You must report the page number in your manuscript where you consider each of the items listed in this checklist. If you have not included this information, either revise your manuscript accordingly before submitting or note N/A.

Reporting Criteria	Reported (Yes/No)	Reported on Page No.
**Reporting of Background**		
Problem definition	Yes	5-7
Hypothesis statement	Yes	7
Description of Study Outcome(s)	Yes	7
Type of exposure or intervention used	Yes	7
Type of study design used	Yes	7
Study population	Yes	5-7
**Reporting of Search Strategy**		
Qualifications of searchers (eg, librarians and investigators)	Yes	8
Search strategy, including time period included in the synthesis and keywords	Yes	8-9
Effort to include all available studies, including contact with authors	Yes	8-10
Databases and registries searched	Yes	8-10
Search software used, name and version, including special features used (eg, explosion)	Yes	8-10
Use of hand searching (eg, reference lists of obtained articles)	Yes	8-10
List of citations located and those excluded, including justification	Yes	8-10
Method for addressing articles published in languages other than English	Yes	8-10
Method of handling abstracts and unpublished studies	Yes	8-10
Description of any contact with authors	Yes	8-10
**Reporting of Methods**		
Description of relevance or appropriateness of studies assembled for assessing the hypothesis to be tested	Yes	8-11
Rationale for the selection and coding of data (eg, sound clinical principles or convenience)	Yes	8-11
Documentation of how data were classified and coded (eg, multiple raters, blinding, and interrater reliability)	Yes	11
Assessment of confounding (eg, comparability of cases and controls in studies where appropriate	Yes	10-11

Reporting Criteria	Reported (Yes/No)	Reported on Page No.
Assessment of study quality, including blinding of quality assessors; stratification or regression on possible predictors of study results	Yes	9
Assessment of heterogeneity	Yes	9
Description of statistical methods (eg, complete description of fixed or random effects models, justification of whether the chosen models account for predictors of study results, dose-response models, or cumulative meta-analysis) in sufficient detail to be replicated	Yes	10-11
Provision of appropriate tables and graphics	Yes	12-18
**Reporting of Results**		
Table giving descriptive information for each study included	Yes	11
Results of sensitivity testing (eg, subgroup analysis)	Yes	13-18
Indication of statistical uncertainty of findings	Yes	11-18
**Reporting of Discussion**		
Quantitative assessment of bias (eg, publication bias)	Yes	18-23
Justification for exclusion (eg, exclusion of non–English-language citations)	Yes	18-23
Assessment of quality of included studies	Yes	18-23
**Reporting of Conclusions**		
Consideration of alternative explanations for observed results	Yes	22-23
Generalization of the conclusions (ie, appropriate for the data presented and within the domain of the literature review)	Yes	22-23
Guidelines for future research	Yes	22-23
Disclosure of funding source	Yes	22-23

Once you have completed this checklist, please save a copy and upload it as part of your submission. DO NOT include this checklist as part of the main manuscript document. It must be uploaded as a separate file.
