# Supplementary material for: A systematic review and meta-analysis on the preventive behaviors in response to the COVID-19 pandemic among children and adolescents
Source: BMC Public Health. 2022 Jun 15;22:1201. doi: 10.1186/s12889-022-13585-z (PMC9200376; doi:10.1186/s12889-022-13585-z)
Supplement: Supplementary file 6 — Additional file 6. R syntax. [file 12889_2022_13585_MOESM6_ESM.docx]

**R syntax**

# load data

> data16 <- read.csv("D:/task/additional work/20210816 meta/compare.csv")

# Load package

library(dmetar)

library(esc)

library(tidyverse)

library(metafor)

# Pre-calculate SMD and the Standard Error

SP_calc <- esc_mean_sd(grp1m = Data16$mean.e, grp1sd = Data16$sd.e, grp1n = Data16$n.e, grp2m = Data16$mean.c, grp2sd = Data16$sd.c, grp2n = Data16$n.c, study = Data16$author, es.type = "SMD") %>%

as.data.frame()

# calculate effect size and heterogeneity

> m.compare <- metagen(TE = es, seTE = se, studlab = Study, data = data16, sm = "SMD", comb.fixed = FALSE, comb.random = TRUE, method.tau = "REML", hakn = TRUE, title = "compare")

> forest.meta(m.compare, layout = "RevMan5")

# Produce eager’s regression test

> metabias(m.compare, method.bias = "linreg")

#pooling effect size of correlation

> dataA <- read.csv("D:/task/additional work/20210816 meta lw/age.csv")

m.cor <- metacor(cor = cor, n = n, studlab = author, data = dataA, comb.fixed = FALSE, comb.random = TRUE, method.tau = "REML", hakn = TRUE, title = "Age")

# Produce forest

> forest.meta(m.cor, layout = "RevMan5")

# Produce funnel plot

funnel.meta(m.cor, xlim = c(-0.5, 2), studlab = TRUE)

#calculate corr effect size and corresponding sample variance

dataG <- read.csv("D:/task/additional work/20210816 meta/gender.csv")

dat <- escalc(measure="ZCOR", ri=r, ni=N, data=data16)

#calculate fail safe - N

fsn(yi, vi, data=dat)

data26 <- read.csv("D:/task/additional work/20210816 meta/adolescents.csv")

fsn(yi, sei, data=dat)

#Produce prediction interval

# Nagashima-Noma-Furukawa prediction interval

# is sufficiently accurate when I^2 >= 10% and K >= 3

PI <- pima(data26$yi, data26$sei, seed = 3141592, parallel = 4)
